# Associations of self-reported skin symptoms with age, sex, and living with a partner: Findings from a representative survey in view of the biomedical and the biopsychosocial model

**DOI:** 10.3389/fmed.2023.1076233

**Published:** 2023-06-21

**Authors:** Hanna Reich, Uwe Gieler, Elmar Braehler, Joerg Kupfer

**Affiliations:** ^1^Institute of Medical Psychology, Justus Liebig University of Giessen, Giessen, Germany; ^2^Depression Research Centre of the German Depression Foundation, Department for Psychiatry, Psychosomatics and Psychotherapy, Goethe University, Frankfurt, Germany; ^3^Department of Dermatology, Justus Liebig University of Giessen, Giessen, Germany; ^4^Department of Psychosomatic Medicine and Psychotherapy, University Medical Centre of Mainz, Mainz, Germany; ^5^Integrated Research and Treatment Centre Adiposity Diseases, Behavioural Medicine Research Unit, Department of Psychosomatic Medicine and Psychotherapy, University of Leipzig, Leipzig, Germany

**Keywords:** skin symptoms, dermatology, cross-sectional studies (MeSH), sociodemographic factors, representative survey, epidemiology, biopsychosocial

## Abstract

**Introduction:**

Social and demographic characteristics are crucial determinants of health. The objective of this contribution is to study the associations of skin symptoms and sociodemographic variables in the general population, and to discuss these findings in view of the biomedical and the biopsychosocial models of skin diseases.

**Methods:**

A national face-to-face household survey with a representative sample of the German general population assessed 19 self-reported skin symptoms (*N* = 2,487). Associations with age, sex, and living situation (alone vs. with partner) were analyzed using logistic regression analyses

**Results:**

The frequencies of pimples and biting of the nails decreased by approximately 30% per age decade, and oily skin, the feeling of disfigurement, excoriations, and sun damages decreased by 8%–15% per age decade. Dryness of the skin increased by 7% per decade. Sensitive skin and dryness were approx. twice as likely in females as in males. Dryness of the skin, itch, and excoriations were 23%–32% more frequent in participants living without a partner.

**Discussion:**

The biomedical model explains some of the findings well (e.g., reduction of pimples with age). The interpretation of other results is facilitated by the biopsychosocial model (e.g., association of living without a partner and itch). This suggests a stronger integration of psychological and social factors into the understanding and treatment of symptoms of the skin.

## Introduction

Social and demographic characteristics are crucial determinants of health and account for avoidable and unjust differences in morbidity and mortality (1). In view of health disparities at national and global levels, sociodemographic indicators became a focus of study in their own right (2). The traditional biomedical model of skin diseases facilitates an understanding of the physical processes associated with sex differences and advancing age in symptoms and morbidity of the skin. The understanding of some skin diseases, however, requires a holistic perspective on the complex interplay between sociodemographic factors and skin morbidity. The biopsychosocial model facilitates focusing on hormonal and other physiological pathways that mediate these interdependencies (3). The objective of this article is to study the associations of skin symptoms and sociodemographic indicators in the general population, and to discuss these findings in view of the biomedical and the biopsychosocial models of skin diseases.

Skin morbidity is associated with age and sex. The skin of younger persons is often affected by acne (4, 5) and its hormonal pathogenesis (6). As time passes, progressive intrinsic ageing (also known as natural or cellular ageing) and the cumulative effects of extrinsic ageing of the skin occur (7). The most characteristic symptoms associated with ageing skin are xerosis and pruritus, which account for as many as 80% of dermatologic complaints among the aged population (8). With regard to sex, findings are mixed regarding the questions who reports skin morbidities and a lifetime history of skin disease more often (9, 10). Fluctuations in female hormone levels have been suggested among the eliciting factors of sensitive skin, including dryness, itch, and redness (11). More scaly skin and eczema have been reported in men (10, 12), while new literature suggests a higher prevalence of psoriasis and acne in women (10, 13).

Skin morbidity may also be associated with other sociodemographic indicators such as social support or socioeconomic status (SES). Social support has been linked to health behavior and psychological processes, beneficial changes in neuroendocrine and immune function, and ultimately to superior general physical and psychological health (14). Skin morbidity (e.g., itch, acne, and hand eczema) has been shown to increase for both genders with poor social support (12, 15, 16). On the other hand, while SES (i.e., income, education, and occupation) is related to morbidity in many health conditions (1), the empirical findings on the social gradient of dermatological symptoms remain ambivalent to this day. Previous studies often reported either no associations (12, 17) or contradictory findings (e.g., higher odds ratios for itch among individuals with a middle household income (12) vs. among individuals with a lower household income (15).

Many of the cited studies focused on a narrow selection of two to six symptoms of the skin. On top of that, some of their associations with sociodemographic indicators seem to represent isolated findings without a theoretical model that was able to integrate potential causal mechanisms explaining these findings. The scope of the present study was to pursue a theory-driven analysis of the associations of 19 self-reported skin symptoms with sociodemographic indicators that were assessed in a representative survey of the general population. For the selection of sociodemographic indicators, we gave preference to those indicators for which the biomedical or the biopsychosocial model offer an explanation about possible causal pathways and for which at least some previous evidence exists. Consequently, age, sex and social support were selected, while education, income, and occupation were not included in the present analyses. This manuscript seeks to answer the following research questions: Is younger age associated with higher frequencies of pimples and blemished skin, while higher age is associated with the reporting of more dryness and pruritus? Is female sex and the absence of social support associated with the reporting of more skin complaints? To operationalize social support for the present analyses based on sociodemographic indicators, we used the information whether a participant was living with a partner or not.

## Methods

### Study design

The current work uses a cross-sectional study design, conducting a self-report assessment of skin symptoms in a representative sample of the German general population The survey was in concordance with the Declaration of Helsinki, and met ethical guidelines of the international code of Marketing and Social Research practice by the International Chamber of Commerce and the European Society for Opinion and Marketing Research. It was approved by the ethics committee of the University of Leipzig (reference: AZ 044–15-09032015).

### Data sampling

A nation-wide face-to-face household survey was conducted in 2015. To recruit a sample that was representative of the noninstitutionalized German general population regarding age, gender, and education, a random sample of the German general population was selected with the assistance of a demographic consulting company (USUMA, Berlin, Germany). The random-route-technique was applied, i.e., random selection of street, house, flat, and target person in the household. If not at home, a maximum of three attempts were made to contact the selected person. The inclusion criteria were a minimum age of 14 years and sufficient knowledge of the German language. After providing written informed consent, self-report questionnaires were presented. All assessments were conducted in the private homes of the participants by trained research assistants. In a first attempt, a sample of 4,844 subjects based on 258 sample points was addressed. In total, 2,513 assessments were conducted (response rate 51.9%).

### Instruments

Sociodemographic variables were obtained and several standardized psychological and sociological questionnaires were filled in. Based on the selection of skin symptoms assessed in a previous survey in 1998 (18), 19 symptoms that are very commonly reported in clinical practice (consensus of UG and JK) were assessed via self-report. Those symptoms were (in alphabetical order): Biting of the nails, body odor, burning sensation of the skin, changes of the skin, dandruff, dryness of the skin, feeling of disfigurement, erythema on the body and on the face, excoriations, ingrown hairs, itch, itchy scalp, oily skin, picking of the cuticles, pimples, sensitive skin, sun damages, and tingling of the skin. Answering options were based on the Giessen Subjective Complaints List (19) and the opening question “To what extent do you currently feel affected by the following complaints? “was answered on a five-point Likert scale (0 = never, 1 = mild, 2 = moderate, 3 = considerable, 4 = severe).

### Statistical analyses

Of the 2,513 interviews conducted, the information about one or more of the predictor variables was incomplete for 16 cases. These cases were excluded from the analyses, as well as another 10 cases that had not responded to *any* item regarding skin symptoms. The continuous age variable was changed to age groups of a decade (<20 years, 20–29 years, 30–39 years, […], ≤80 years) to facilitate interpretation of results. Sex (male/ female) and living with a partner (no/ yes) entered as dichotomous variables into the analyses. Significant intercorrelations (Spearman) between predictor variables were reported. Response categories of the items about skin symptoms were dichotomized to distinguish between ‘cases’ and ‘non-cases’ (answering option ‘0-never’ vs. answering options ‘1-mildly’ to ‘4-severely’). Data were weighted to improve representativeness regarding age, sex, and education. Multiple logistic regression analyses were carried out to test for the associations of each skin symptom with age, sex, and living with a partner. Each outcome represented an independent set of hypotheses of the associations between the respective skin symptom and sociodemographic indicators and was tested with alpha = 0.05. Odds ratios (ORs) and 95% confidence intervals for ORs are given. R version 3.5.2 and Stata version 15.1 were used to conduct statistical analyses (20, 21).

## Results

### Sample characteristics

The analysed sample consisted of *N* = 2,487 subjects. 55.5% were female and the mean age was 48.9 ± 18.0 years (age range: 14–94 years). 96.5% of the participants were holding the German nationality and 66.0% had completed 10 or more years of education. 40.0% were working full-time, 15.0% were working part-time, and 27.2% of the participants were pensioners. In the present sample, higher age was associated with a higher frequency of living with a partner (*r* = 0.07, *p* < 0.001) and female sex was associated with a lower frequency of living with a partner (*r* = −0.09, *p* < 0.001).

### Skin symptoms associated with Age

The skin symptoms that were most common (point prevalence rates >30% in one or both sexes) in the younger age groups (14–19 and 20–29 years) were: biting of the nails, body odor, changes of the skin, dandruff, dryness, excoriations, itch, itchy scalp, oily skin, pimples, and sensitive skin. Of these, pimples were reported by more than 50% of all respondents aged 14 to 29 years (see Table 1 for point prevalence rates). With increasing age, the frequencies of pimples and biting of the nails decreased by approximately 30% per decade, and the frequencies of an oily skin, the feeling of disfigurement, excoriations, and sun damages decreased by 8%–15% per decade (see Table 2 for ORs). In the older age groups (70–79 and 80–94 years), the most common skin symptoms were: body odor, changes of the skin, dandruff, dryness, itchy scalp, and sensitive skin. Dryness of the skin increased by 7% per decade.

**Table 1:** Point prevalences (%) of 19 self-reported skin symptoms by age group and sex.

	Sex	*N*	Age group								Overall
			14–19	20–29	30–39	40–49	50–59	60–69	70–79	80–94	
			106	353	376	421	463	400	289	79	2,487
Biting of the nails	Female	1,366	24.60	17.65	10.56	10.85	10.01	3.75	2.25	1.85	9.58
	Male	1,100	32.25	21.57	14.41	9.84	12.82	4.15	2.01	13.95	12.44
Body odor	Female	1,366	29.05	21.29	31.41	22.11	32.41	26.26	24.30	20.06	26.08
	Male	1,098	34.79	31.51	25.37	33.26	25.85	32.90	33.30	19.64	30.28
Burning sensation	Female	1,369	18.34	13.88	10.67	14.22	15.18	11.48	20.09	13.36	14.80
	Male	1,097	17.06	7.76	9.53	11.67	13.43	13.01	10.43	10.71	11.46
Changes of the skin	Female	1,371	40.69	27.16	24.84	29.98	29.99	28.25	33.75	25.64	29.87
	Male	1,095	29.03	25.00	14.27	15.26	18.77	32.78	26.96	20.56	21.82
Dandruff	Female	1,368	25.79	26.63	22.66	26.52	21.51	17.39	19.34	20.56	22.51
	Male	1,100	38.09	36.80	35.03	35.78	26.32	34.90	39.19	11.86	34.08
Dryness	Female	1,373	38.13	48.54	40.83	49.25	49.29	53.81	54.46	59.32	49.37
	Male	1,100	30.63	42.50	31.33	28.00	28.82	43.28	37.21	33.41	34.03
Erythema (body)	Female	1,370	25.59	29.64	19.96	29.23	29.15	20.22	28.05	25.01	26.31
	Male	1,096	22.90	15.15	14.15	20.13	14.25	25.50	22.50	22.02	18.70
Erythema (face)	Female	1,368	25.50	24.41	19.20	24.42	24.98	19.86	26.85	23.20	23.72
	Male	1,098	21.81	16.33	14.55	16.39	15.29	23.49	17.69	12.05	17.20
Excoriations	Female	1,367	13.44	17.95	14.77	11.86	16.06	5.93	9.93	15.39	12.93
	Male	1,096	32.81	21.14	13.93	8.39	15.64	15.00	11.78	13.70	15.22
Feeling of disfigurement	Female	1,367	13.82	7.51	6.14	4.75	7.31	2.18	3.49	2.10	5.60
	Male	1,097	2.12	5.50	6.06	5.40	5.51	4.18	2.00	7.77	4.76
Ingrown hairs	Female	1,368	8.41	8.58	10.63	7.40	9.43	8.42	7.47	14.36	8.89
	Male	1,098	9.55	24.04	13.67	13.81	11.71	18.61	14.52	16.64	15.29
Itch	Female	1,373	24.22	30.74	23.19	26.59	31.46	25.03	27.76	28.22	27.42
	Male	1,099	28.75	25.10	15.18	20.17	20.57	26.50	22.70	19.16	21.84
Itchy scalp	Female	1,367	21.98	27.42	23.68	35.18	31.24	28.50	32.69	32.44	29.85
	Male	1,097	32.09	25.83	20.27	27.67	23.74	27.07	32.95	27.97	26.58
Oily skin	Female	1,370	49.74	27.62	22.49	20.51	19.59	18.54	18.00	13.48	22.33
	Male	1,098	41.99	29.39	23.64	18.05	21.21	19.51	16.00	13.51	22.32
Picking of the cuticles	Female	1,361	18.28	19.14	14.36	17.81	22.61	13.02	15.57	17.88	17.36
	Male	1,098	23.87	17.10	14.07	12.78	14.21	16.51	15.87	23.69	15.67
Pimples	Female	1,367	64.16	61.30	36.77	40.06	34.17	17.77	14.05	10.00	34.03
	Male	1,097	68.09	49.58	35.77	26.38	26.20	21.57	12.40	13.70	30.88
Sensitive skin	Female	1,364	45.10	46.91	39.23	49.06	49.25	46.28	42.52	41.64	45.40
	Male	1,094	23.61	32.07	26.35	27.00	20.46	30.40	27.53	16.64	26.45
Sun damaged skin	Female	1,361	28.56	24.44	17.79	21.96	22.24	18.44	13.88	9.24	19.66
	Male	1,093	17.56	20.90	15.50	13.47	13.39	20.26	17.36	13.70	16.44
Tingling of the skin	Female	1,371	18.01	13.87	12.58	19.51	22.46	19.84	20.47	22.77	18.67
	Male	1,099	20.53	18.05	12.49	10.94	15.95	17.17	13.61	17.75	14.94

Frequencies of participants answering with 1–4 are given, i.e., “yes, I’m affected mildly, moderately, considerably, or severely by this complaint.” Symptoms are ordered alphabetically. Data were weighted regarding age, sex, and education to improve representativeness.

**Table 2:** Odds ratios for 19 self-reported skin symptoms in relation to age, sex, and living with a partner.

	*N*	Age (rise per 10 years)		Sex (reference: male)		Living with a partner (reference: no)	
		OR [95% CI]	*p*	OR [95% CI]	*p*	OR [95% CI]	*p*
Biting of the nails	2,466	**0.68 [0.62; 0.75]**	**<0.001**	0.79 [0.58; 1.06]	0.118	1.15 [0.85; 1.55]	0.374
Body odor	2,464	0.99 [0.94; 1.04]	0.712	**0.81 [0.67; 0.99]**	**0.041**	0.97 [0.79; 1.19]	0.757
Burning sensation	2,466	1.03 [0.96; 1.11]	0.443	**1.32 [1.01; 1.74]**	**0.043**	0.91 [0.70; 1.20]	0.532
Changes of the skin	2,466	1.03 [0.97; 1.08]	0.360	**1.49 [1.21; 1.84]**	**<0.001**	0.81 [0.66; 1.004]	0.055
Dandruff	2,468	0.93 [0.90; 1.002]	0.058	**0.57 [0.46; 0.70]**	**<0.001**	0.98 [0.80; 1.21]	0.853
Dryness	2,473	**1.07 [1.02; 1.13]**	**0.005**	**1.82 [1.51; 2.19]**	**<0.001**	**0.73 [0.61; 0.88]**	**0.001**
Erythema (body)	2,466	1.03 [0.97; 1.09]	0.387	**1.54 [1.24; 1.92]**	**<0.001**	0.98 [0.78; 1.22]	0.831
Erythema (face)	2,466	1.02 [0.96; 1.08]	0.625	**1.48 [1.18; 1.86]**	**<0.001**	0.92 [0.73; 1.15]	0.446
Excoriations	2,463	**0.91 [0.85; 0.98]**	**0.008**	0.82 [0.64; 1.07]	0.146	**0.68 [0.52; 0.88]**	**0.004**
Feeling of disfigurement	2,464	**0.88 [0.79; 0.97]**	**0.010**	1.21 [0.81; 1.81]	0.349	0.80 [0.54; 1.18]	0.256
Ingrown hairs	2,466	1.00 [0.92; 1.08]	0.948	**0.53 [0.40; 0.71]**	**<0.001**	0.86 [0.65; 1.15]	0.312
Itch	2,472	1.01 [0.96; 1.07]	0.680	**1.32 [1.07; 1.63]**	**0.009**	**0.77 [0.63; 0.95]**	**0.016**
Itchy scalp	2,464	1.05 [0.999; 1.11]	0.055	1.15 [0.94; 1.40]	0.184	0.88 [0.71; 1.07]	0.203
Oily skin	2,468	**0.85 [0.79; 0.90]**	**<0.001**	1.03 [0.83; 1.29]	0.771	0.85 [0.68; 1.06]	0.148
Picking of the cuticles	2,459	0.98 [0.92; 1.05]	0.563	1.13 [0.89; 1.44]	0.323	0.93 [0.73; 1.19]	0.579
Pimples	2,464	**0.68 [0.64; 0.72]**	**<0.001**	**1.28 [1.08; 1.57]**	**0.016**	0.91 [0.75; 1.12]	0.377
Sensitive skin	2,458	0.99 [0.94; 1.04]	0.683	**2.29 [1.89; 2.78]**	**<0.001**	0.84 [0.69; 1.02]	0.083
Sun damaged skin	2,454	**0.92 [0.86; 0.98]**	**0.012**	**1.28 [1.01; 1.62]**	**0.043**	1.09 [0.86; 1.38]	0.473
Tingling of the skin	2,470	1.04 [0.97; 1.11]	0.239	**1.28 [1.007; 1.62]**	**0.043**	0.89 [0.70; 1.13]	0.328

Significant associations are printed in bold.

### Skin symptoms associated with sex

The most common skin symptoms in females were: dryness, pimples, and sensitive skin. Sensitive skin and dryness were approximately twice as likely in females as in males. Furthermore, female sex was associated with higher frequencies of burning sensations, changes of the skin, erythema on the face and on the body, itch, pimples, sun damages, and tingling of the skin. The most common skin symptoms in males were: body odor, dandruff, dryness, and pimples. Male sex was associated with statistically significant higher frequencies of dandruff, ingrown hairs, and body odor.

### Skin symptoms associated with living with a partner

Participants living without a partner were 23%–32% more likely to report about dryness of the skin, itch, and excoriations (see Table 2).

## Discussion

In the following, the reported associations between skin symptoms and sociodemographic indicators will be discussed in view of the literature and with respect to the biomedical and the biopsychosocial model of skin diseases. As expected (4, 5) and in line with the biomedical model of skin symptoms, the present analyses showed an association of younger age with pimples and an oily skin, reflecting the hormonal pathogenesis of acne in younger age. Associations of psycho-behavioral skin symptoms (i.e., biting of the nails, excoriations, sun damages, feeling of disfigurement) with younger age have been reported before (22–25). The understanding of these associations might be facilitated by the biopsychosocial model, as it takes mediating and moderating influences of cognitive, emotional, and behavioral responses to external stressors and age-related changes into account (3). As hypothesized, dryness of the skin, a core symptom of intrinsic ageing, was associated with higher age. Structural and physiologic changes of the skin occur as a natural consequence of intrinsic and extrinsic ageing (7, 8). This concurs with the clinical phenomenon of senile xerosis that appears due to physiologic changes of ageing skin: reduced movement of water from dermis to epidermis leads to reduced epidermal hydration, and reduced stratum corneum lipids cause a decreased ability to retain water (8). In the present analysis, higher age was not associated with itch. Various other studies, including the classic Lambeth study, found no associations between age and ‘prurigo and allied conditions’ (26), prevalence rates of itchy rash (9), or current chronic pruritus and pruritus within the last 12 months (17). The empirical basis seems, however, inconsistent as some studies also reported that *acute* itch decreased with age (12, 15), while on the other hand, a positive relationship between *chronic* pruritus and age has been found (27, 28).

As expected, women were affected by more skin symptoms than men, a result that seems to be consistent also among different ethnic groups (29). In line with previous studies, women experienced more sensitive skin than men (30), were more likely to suffer from acute as well as chronic pruritus (12, 15, 17), and had a higher risk of being affected by pimples (12). ORs were comparable to those reported previously (4). Sensitive skin, dryness, erythema, itching, and pimples are associated with female hormone fluctuations (11). The biomedical and the biopsychosocial model help explaining the higher prevalence of these symptoms in women, as cyclically fluctuating levels of estrogen and progesterone influence lipid secretion, skin hydration, and barrier function (31), as well as emotional and behavioral responses to the menstrual cycle that may, in turn, affect skin symptoms (32). The results regarding the association between male sex and a higher prevalence of dandruff and ingrown hairs are congruent with studies that found more scaly skin, psoriasis, and eczema in men (12, 26). However, while in the current analysis men reported about body odor more frequently than women, sweating has been reported before to be more prominent in women than in men (4). This divergence might be explained by different perception and reporting of ‘sweating’ and ‘body odor’ between genders.

Participants living without a partner were more likely to be affected by dry skin, itch, and excoriations, which is consistent with previous studies (12, 15). These finding may be explained using the biopsychosocial model: Marital quality can affect biological mediators such as neuroendocrine axes and immune function (33), which are in turn linked to surrogate and clinical endpoints of skin symptoms and disease. Studies of married couples’ interaction gave a hint to possible pathways from social interactions to skin morbidity: More positive and fewer negative communications were associated with higher oxytocin and vasopressin levels and with faster wound healing (34). On the contrary, hostile interactions were associated with changes in proinflammatory cytokine production and slower wound healing (35). Meta-analytic findings showed that the effect sizes for the association of marital quality and health in general are small, but similar in magnitude to those of other health behaviors (e.g., diet) and health outcomes (36).

### Strengths and limitations

The present study examined a very broad spectrum of 19 skin symptoms and was based on a representative sample of the German general population covering a wide age range, including participants from 14 to 94 years. Nonetheless, there are some limitations to address. Living with a partner can be an important proxy of social and partnership support, but the dichotomous item did not provide any information about the quality of the partnership. Social support is usually defined by both, the structure of an individual’s social ties and the explicit functions they may serve (14). The associations of skin symptoms with support from family, friends, and colleagues, as well as the quality of these contacts require further study. The item ‘living with a partner’ might have been especially problematic in the very young and in the very old participants, where other living arrangements offering social support (e.g., living with parents, living in a retirement home) are common, but have not been analyzed in the present study. Other, possibly relevant sociodemographic indicators such as ethnicity (29), residential area (17, 30), or country of residence (9) were also not addressed here. While there was at least some evidence in the literature about the associations between the sociodemographic indicators that were assessed in the current manuscript and skin symptoms, the present analyses still have explorative character and as such, shall be understood as a hypothesis-generating contribution rather than confirmatory testing. The burden of skin symptoms for each participant was assessed via self-report. Self-report data represent the individual experience of a problem and are subject to reporting biases. That means the findings cannot be applied to skin diseases without further ado, since this study did not include an evaluation of skin conditions by a medical doctor or an evaluation of medical records.

## Conclusion

Taken together, the present analyses reinforce the high importance of a holistic view of a person presenting with symptoms of the skin. Most of the assessed symptoms were associated with one or more of the sociodemographic indicators and many of these associations were not explainable by a biomedical model alone. This suggests a stronger integration of psychological and social factors into the understanding and treatment of symptoms of the skin. Future research needs to integrate the mediating and moderating effects of external factors (e.g., stress) via internal factors (such as emotional reactions and cognitive appraisal) on the autonomic nervous system, neuroendocrine axes, and immune function to recognize critical mechanisms and to address them properly in the multidisciplinary treatment of skin diseases. Based on our results, we advocate for a stronger integration of psychological and social factors into the understanding and treatment of symptoms of the skin.

## Data availability statement

The datasets presented in this article are not readily available because they are only a subpart of a bigger dataset. Requests to access the datasets should be directed to HR, hanna.reich_de_paredes@deutsche-depressionshilfe.de.

## Ethics statement

The studies involving human participants were reviewed and approved by Ethics Committee of the University of Leipzig (reference: AZ 044–15‐09032015). Written informed consent to participate was provided by all participants or the participants’ legal guardian/next of kin.

## Author contributions

JK, UG, and EB contributed to conception and design of the study. EB organized the database. HR performed the statistical analysis and wrote the first draft of the manuscript. HR, EB, and JK contributed to manuscript revision. All authors contributed to the article and approved the submitted version.

## Conflict of interest

The authors declare that the research was conducted in the absence of any commercial or financial relationships that could be construed as a potential conflict of interest.

## Publisher’s note

All claims expressed in this article are solely those of the authors and do not necessarily represent those of their affiliated organizations, or those of the publisher, the editors and the reviewers. Any product that may be evaluated in this article, or claim that may be made by its manufacturer, is not guaranteed or endorsed by the publisher.
